# Comparative genomic hybridization analysis of invasive ductal breast carcinomas in the Chinese population

**DOI:** 10.3892/ol.2015.3608

**Published:** 2015-08-14

**Authors:** JIANWEI ZHANG, HONGYAN ZHANG, XIN XU, MINGRONG WANG, ZHONGHE YU

**Affiliations:** 1 Department of Oncology, Beijing Army General Hospital, Beijing 100700, P.R. China; 2 National Laboratory of Molecular Oncology, Cancer Institute, Chinese Academy of Medical Sciences and Peking Union Medical College, Beijing 100021, P.R. China

**Keywords:** comparative genomic hybridization, ductal breast carcinomas, copy number changes

## Abstract

Breast cancer is the most common malignancy in Chinese women. The aim of the present study was to investigate the genetic alterations that occur in breast cancer cells in Chinese women. Comparative genomic hybridization (CGH) analysis was performed on 34 tumors obtained from patients with primary invasive ductal breast carcinoma (IDC). Recurrent genetic alterations in breast cancer include gains on chromosomes 1q (59%), 16p (50%), 17q (44%), 8q (38%), 11q (32%), 20q (32%), 1p (24%), 20p (24%), 19q (21%) and 19p (18%). Losses are common on chromosomes 6q (15%), 8p (12%), 18 (12%), 4q (9%), X (9%) and 17p (9%). In the present study, high-level amplifications were observed on chromosomes 1q32, 8p, 11q13, 17q and 20q. Overall, the chromosomal DNA gains observed were consistent with the changes reported in Caucasian populations. However, the incidence of chromosomal DNA loss was lower in the present study compared with the incidence reported in the literature. The present results demonstrate the pattern of chromosomal imbalances in the invasive ductal breast carcinomas of Chinese females.

## Introduction

Comparative genomic hybridization (CGH) is a molecular cytogenetic technique that is based on measuring the relative fluorescent hybridization intensities of two genomic complexity hybridization probes (1). CGH is used to detect changes in the copy number of specific chromosomes or chromosomal regions, such as changes in the relative genome size and ploidy level in test samples, by comparing the samples with reference DNA. This technique was originally developed for the evaluation of differences in chromosomal complements between solid tumors and normal tissues. CGH demonstrates an improved resolution when compared with traditional cytogenetic analysis techniques, including Giemsa banding patterns or fluorescence *in situ* hybridization (FISH), and possesses the potential to detect changes of 5–10 megabases, which is also superior to traditional cytogenetic analysis techniques (2). CGH was the first efficient approach for scanning the entire genome for DNA copy number variations in a single experiment. In a typical CGH measurement, total genomic DNA is isolated, which is then globally assayed for the detection of chromosomal gains and losses in solid tumors. The application of CGH to DNA extracted from tumor specimens has revealed the chromosomal map position of DNA gains and losses by comparing the extracted DNA with reference metaphase preparations under high resolution (3). CGH databases from several studies of the same tumor type revealed consistent patterns of non-random genetic aberrations (4). Certain changes appeared to be common to various types of malignant tumors, while others were more tumor-specific. The main disadvantage of array CGH is the limited ability of the method to detect aberrations that do not result in copy number changes and mosaicism (5).

Breast cancer currently ranks as the most common malignancy among women in China. Despite far more studies on the genetic alterations in breast cancer being reported in Caucasian populations, little is known about the etiology of breast cancer in the Chinese population (6). In total, 30 susceptibility gene loci have been identified in breast cancer. Among these genes, several rare mutations confer a considerably increased genetic risk for breast cancer. However, numerous mutations are widespread in patients with a slightly increased risk of breast cancer. Overall, however, these alleles explain <30% of familial breast cancer risk (7,8). Therefore, CGH was utilized in the present study to analyze 34 female Chinese patients with primary IDC of the breast to identify specific gene mutations.

## Materials and methods

### 

#### Tumor samples

Tumor tissue samples were obtained from 34 patients with primary invasive ductal carcinoma (IDC) of the breast at the Cancer Hospital of the Chinese Academy of Medical Sciences (Beijing, China), between February 1997 and August 1999. The histopathological diagnosis was confirmed independently by at least two experienced pathologists. The tissues were stored at −80°C prior to DNA extraction. The DNA was extracted using an equal volume of phenol/chloroform/isoamylalcohol extraction and digested using proteinase K. The detailed clinicopathological data for the enrolled patients is summarized in Table I. This study was approved by the ethics committee of the Beijing Army General Hospital (Beijing, China) and written informed consent was obtained from all patients.

#### Comparative genomic hybridization

Reference metaphase cells were prepared from the phytohemagglutinin (PHA)-stimulated peripheral blood lymphocytes of a normal donor using standard cytogenetic procedures (9). The slides were treated with 100 µg/ml RNase in 2X saline-sodium citrate (SSC) buffer and 50 µg/ml pepsin in 0.01 N HCl. Tumor DNA was labeled by nick translation with Biotin-16-deoxyuridine triphosphate (dUTP), and reference DNA obtained from placenta (Invitrogen Life Technologies, Carlsbad, CA, USA) was labeled using digoxigenin-11-dUTP (Boehringer Ingelheim, Ingelheim, Germany). The final size of the labeled fragments was 600–1500 bp (10).

Equal quantities of the tumor and reference DNA samples (500 ng) were mixed and precipitated with 20 µg unlabeled human Cot-1 DNA (Invitrogen Life Technologies). The DNA samples were dissolved in 5 µl of hybridization mixture, consisting of 50% formamide/2X SSC buffer and 1.25 µl 40% dextran sulfate/1.25 µl ddH_2_O, and denatured for 8 min at 75°C. The metaphase slides were denatured in 70% formamide/2X SSC buffer (pH 7.0) for 3 min at 70°C, and then dehydrated in 70, 85 and 100% ethanol. The hybridization mixture was applied to the slides and the slides were hybridized for 2–3 days at 37°C in a moist chamber.

Subsequent to hybridization, the slides were washed in 50% formamide/2X SSC buffer at 43°C for 15 min. The hybridization signals of the tumor genomic probes were detected by two layers of fluorescein isothiocyanate (FITC)-conjugated avidin and amplified with one layer of monoclonal goat anti-rabbit anti-avidin antibody (1:100; cat. no. VL 201-3215; Vector Laboratories, Inc., Burlingame, CA, USA), while the signals of the reference DNA probes were visualized with antidigoxin Fab fragments conjugated to tetramethylrhodamine isothiocyanate (Sigma-Aldrich, St. Louis, MO, USA). The chromosomes were counterstained with DAPI (Sigma-Aldrich), a solution for chromosome identification.

#### Microscopy and digital image analysis

Gray level images were acquired for each fluorochrome with a cooled charge-coupled device camera (Prinston Pharmaceutical, Inc., Cranbury, NJ, USA) mounted on an Opton fluorescence microscope. Chromosomes were identified using DAPI banding. Excitation of each fluorochrome was accomplished by using single-band-pass excitation filters (Zeiss, Oberkochen, Germany) in a computer-controlled filter wheel (Zeiss). This enabled the collection of correctly registered sequential images of the three fluorochromes, DAPI, fluorescein isothiocyanate and rhodamine. The three-color images were processed using a Metamorph Imaging System (Universal Imaging Corporation, Sunnyvale, CA, USA) for pseudocolor visualization. Contrast-stretched three-color images were used to visually inspect the color changes along the metaphase chromosomes.

A quantitative analysis of the intensity of the green and red fluorescence was performed using CGH Analyzer software (Tsinghua National Laboratory for Information Science and Technology, Beijing, China). Briefly, subsequent to the determination of the chromosomal axis for each metaphase chromosome, individual FITC and rhodamine profiles were calculated. These were used for the computation of the FITC to rhodamine ratio profiles. Gain and loss abnormalities were defined by setting the thresholds at 1.2 and 0.8, respectively (11). The ratio profiles were computed as the mean values of at least five metaphase spreads.

## Results

**Figure 1.**
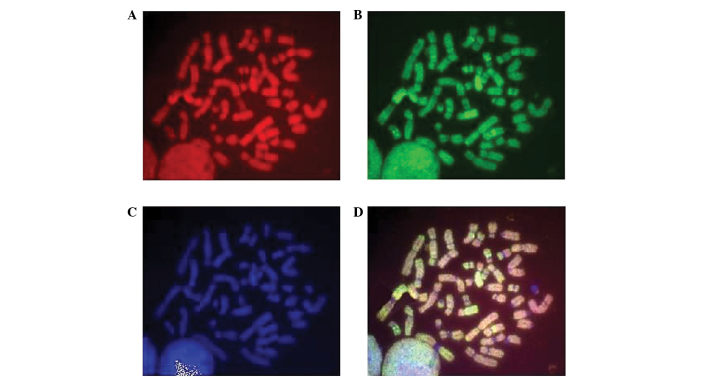
Fluorescence photomicrograph revealing the results of comparative genome hybridization of tissue from invasive ductal carcinoma. (A) DNA extracted from tumor tissues was labeled in green and (B) normal reference DNA was labeled in red. (C) A normal metaphase chromosome was counterstained blue with DAPI. (D) The tumor and normal DNA were hybridized to the normal metaphase chromosome. Chromosomal regions that were over-represented in the tumor exhibit a predominantly green color, whereas regions possessing deletions in the tumor demonstrate a predominantly red color. The overlap represents the ratio of copy number changes between the tumor and control DNA.

**Figure 2.**
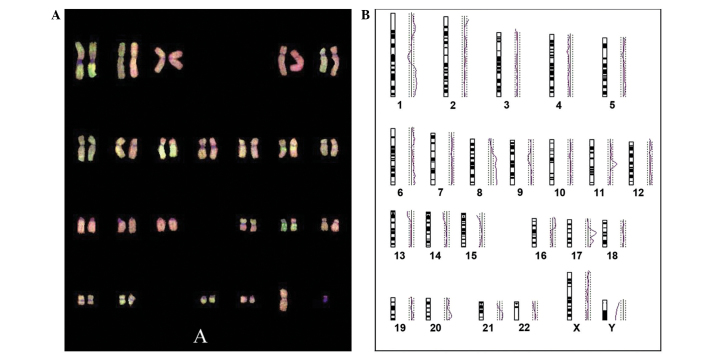
(A) Karyotype images and (B) ratio profiles. The three vertical lines on the right of the chromosome ideograms represent the various values of the fluorescence intensities between the tumor and reference genome. The values are 0.8, 1.0 and 1.2 from left to right, respectively.

**Figure 3.**
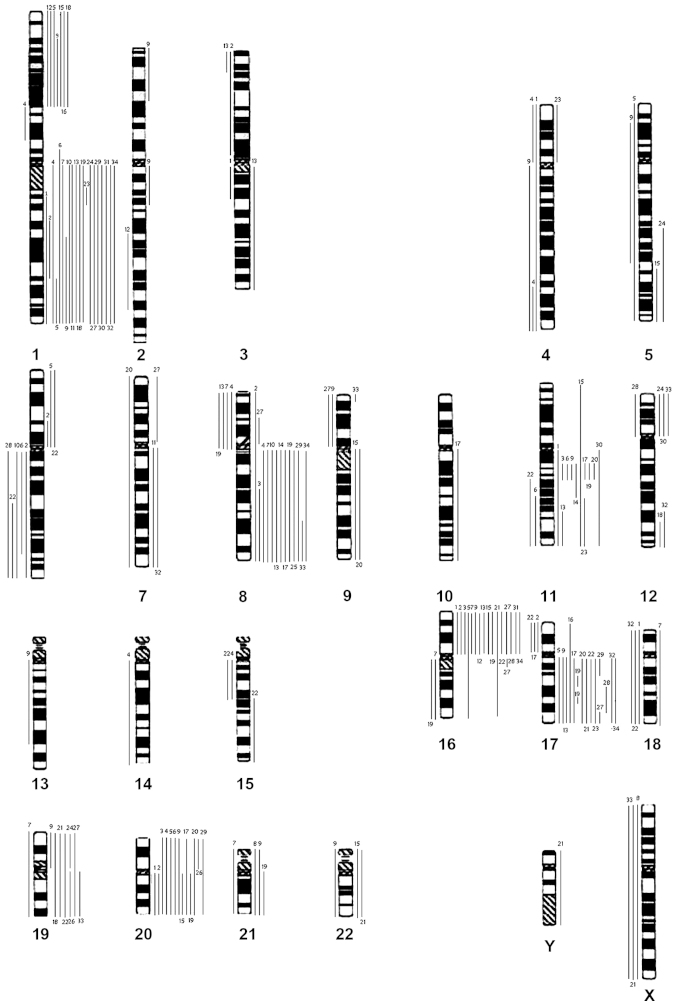
Summary of the genetic imbalances detected by comparative genome hybridization in 34 invasive ductal breast carcinoma tumor samples. Vertical lines on the left side of each chromosome ideogram represent a loss of genetic material in the tumor, whereas those on the right correspond to a gain. The numbers above the bars refer to the case numbers.

All 34 patients with primary IDCs possessed DNA-sequence copy number variations that involved one or more regions of the genome (Table II; Figs. 1 and 2). An overview of the gain and loss of chromosomal material detected in the present of 34 tumors is summarized in Fig. 3.

Overall, recurrent gains of DNA copy number regions occurred on chromosome 1q in 20 out of 34 tumors (59%), 16p in 17 out of 34 tumors (50%), 17q in 15 out of 34 tumors (44%), 8q in 13 out of 34 tumors (38%), 11q in 11 out of 34 tumors (32%), 20q in 11 out of 34 tumors (32%), 1p in 8 out of 34 tumors (24%), 20p in 8 out of 34 tumors (24%), 19q in 7 out of 34 tumors (21%) and 19p in 6 out of 34 tumors (18%).

High-level amplifications were observed on chromosomes 1q3, 8q, 11q13, 17q and 20q. Regions of losses were identified on chromosome 6q in 5 out of 34 tumors (15%), 8p in 4 out of 34 tumors (12%), 18 in 4 out of 34 tumors (12%), 4q in 3 out of 34 tumors (9%), X in 3 out of 34 tumors (9%), and 17p in 3 out of 34 tumors (9%) (Table III).

## Discussion

IDCs of the breast account for 80% of all breast cancers, and demonstrate a worse survival rate than invasive lobular carcinomas (ILCs) (12). In the present study, CGH analysis revealed that 34 patients with IDC of the breast possessed complicated chromosomal imbalances. As aforementioned, gain of DNA sequences most frequently involved the chromosomes and chromosomal regions of 1q, 16p and 17q. Several studies have employed CGH to detect chromosomal imbalances in breast cancers and reported frequent genetic aberrations, the majority of which were consistent with the present findings (13–15).

CGH enables the rapid detection and mapping of the increases and decreases in the copy number of DNA sequences at any location in the tumor genome, providing an overview of the copy number changes that occur in solid tumors (12,16,17). Although cytogenetic analysis does provide a similar overview to CGH, cytogenic analysis is limited by technical issues in preparing metaphase chromosomes from solid tumors, the inability to determine the genomic origin of the amplified sequences, such as homogeneously stained regions and double-minute chromosomes, and difficulties in the unambiguous identification of all changes in highly aberrant genomes.

The present results contribute to the current knowledge on the frequency and chromosomal distribution of DNA gains and losses in breast cancer. Previous studies have demonstrated that frequent gains occur on chromosomes 1q, 8q, 11q, 16p, 17q, 20q and 19. Losses have been revealed to be common on chromosomes 6q, 17p, 16q, 22q and 3p. Several studies have provided evidence that loss or deletion on chromosome 17p12-13 is associated with chemotherapy resistance in breast cancer, as well as esophageal, lung, liver and stomach cancer (1,2,5,7–10). A study performed in Brazil that investigated only two patients demonstrated loss of considerable portions of chromosomes 17, 19 and 22 (18). The findings of this previous study are consistent with the present results. Furthermore, novel regions of gains or losses, such as 11q and 4 q, were also identified in the present study. Among the chromosomal regions containing gains and losses were numerous genes that were important for the progression of breast cancer. The sites of localized high-level DNA amplification harboring known oncogenes identified in the present study consisted of 7p12 (*EGFR*), 8q24 (*MYC*), 11q13 (*CCND1*), 12q14 (*MDM2*), 17q12 (*ERBB2*), 20q12 (*AIB1*) and 20q13 (*ZNF217*) (Table III). Deletions within known tumor suppressor genes identified in the present study consisted of 13q12 (*BRCA2*), 17p13 (*TP53*) and 17q21 (*BRCA1*) (19). The gene located at 17p12 may be associated with the clinical outcomes of chemotherapy. Kim *et al* revealed that 17p12 loss was more frequent in the ovarian carcinoma patients with chemoresistant serous ovarian carcinoma than those with chemosensitive lesions (20). Han *et al* used microarray CGH to reveal the existence of a significant association between deletion of 17p12 and resistance to chemotherapy in breast cancer (21). Sun *et al* reported that a copy number variation at 16q22.1 in breast cancer is a frequent event (22). A study performed using 11 primary breast cancers and their matched lymph node metastasis lesions revealed gains on chromosomes 1q, 8q and 17q and losses on chromosomes 6q, 8p, 9q, 13q, 16q, 17p and Xp (23). A CGH study using 18 breast cancer cell lines revealed extensive DNA copy number changes. All cell lines possessed a gain at 8q22-qter with changes also occurring at 1q31-32, 20q12-q13.2, 8q13, 3q26.1-qter, 17q21-qter, 5p14, 6p22 and 22pter-qter. Furthermore, cytogenetic studies have identified gains on 8q, 17q12 and 20q13 that are associated with poor overall survival rate of breast cancer (24).

The majority of studies in the literature have been performed in Caucasian or Mediterranean populations and extremely few details are available on the genetic modifications in Asian populations with breast cancer (25,26). A CGH analysis was performed in Germany using a cohort of 105 patients with IDC. This study demonstrated that IDC tissues possessed an increased number of alterations compared with ILC tissues, providing a genetic correlation to the overall association between ductal carcinoma and a worse prognosis compared with ILC (27). Another study performed in Greece using a cohort of eight patients with primary breast cancer revealed recurring regions of gain on chromosomes 1q, 20q and 8q, while the most common regions of loss were on chromosomes 3p and 6q (28). A CGH analysis of 40 pT2 tumors excised from breast cancer patients of a racially homogenous population in southern China revealed a complex pattern of genetic alteration, with the most frequent chromosomal gains identified on chromosomes 1q, 8q, 11q13, 16p, 17q and 20q, and frequent losses on chromosomes 8p, 11q, 13q and 18q (29). When compared with previous studies, the chromosomal DNA changes observed in the present study were consistent with the changes reported in the Caucasian population and in the previous study of an Asian population. However, the incidence of chromosomal DNA losses was lower than previously reported (30). This may be due to the deletions of particular genes not being universal in the IDC tissues of Chinese women and CGH technology is also not sensitive enough to detect smaller chromosome DNA losses (1,2).

The present data obtained by CGH define a series of genomic imbalances, which are a characteristic feature of multiple IDCs. The current results provide candidate regions for potential oncogenes and tumor suppressor genes associated with breast cancer in the Chinese population, a subject that requires investigation in future studies.

## Figures and Tables

**Table I.** Clinicopathological data on the 34 invasive ductal carcinomas.

Characteristic	Value
Average age, years	49.2
Stage, n	
I	2
II	18
III	12
IV	2
Grade, n	
1	4
2	19
3	11

**Table II.** Chromosomal gains and losses in 34 invasive ductal breast carcinoma tumors, detected by comparative genomic hybridization.

Case	Gain	Loss
1	1p3, 1q2-43, 11q, 16p, 20q	3q1, 4, 18
2	1p3, 1q24-32, 6p13, 8, 16p, 20q	3p, 17p, 6q
3	8q1, 11q13, 16p, 20	
4	1q, 8q, 20	1p2, 4p, 4q3, 5, 8p, 14q, 15q1
5	1p3, 1q33-ter, 6p, 16, 17q, 20	18
6	1p12-1qter, 11q13, 16p, 20	6q11-24, 11q2
7	1q, 8q, 16p, 18	8p, 16q, 19, 21
8	21	X
9	1p31-35, 1q3-ter, 2p2-ter, 2q1, 11q13, 16p, 17q, 19p	4q, 5p14-5q23, 9p, 13q1-31
10	20, 21	22
11	1q, 8q	6q
12	1q, 7q	
13	16p	2q23-34
14	1q, 3q, 8q, 11q23-ter, 16p, 17q	3p25-ter, 8p
15	8q, 11q13-14	
16	1p3, 5q3, 9q, 11, 16p, 20q, 22	
17	1p3, 17	
18	8q, 10q, 11q13, 17q, 20	17p
19	1p3, 1q, 12q24, 19	
20	1q, 8q, 11q13, 16p, 17q21, 17q23, 20q, 21q	8p, 16q
21	9q, 11q13, 17q, 20	17
22	16, 17q, 19, 22, Y	X
23	6p, 15q2, 16p, 17q, 19	6q2, 11q14-ter, 15q1, 17p, 18
24	1q21, 4p, 11q2, 17q	
25	1q, 5q2-ter, 12p, 19p	
26	8q	
27	19q, 20p	
28	1q, 7p, 8p1, 16p, 19, 16q11, 17q25	9p
29	16p, 17q22-24	6q, 12p
30	1q, 8q, 17q1, 20	
31	1q, 11q, 12p	
32	1q, 16p	
33	1q, 7q, 12q23-ter, 17q	18
34	8q23-ter, 9p24, 12p1, 19q, 1q, 8q, 16p, 17q	X

**Table III.** Chromosome arms with frequent gains and loses (n=34).

Locus that contains increased copy numbers	Frequency, n (%)	Locus that contains decreased copy numbers	Frequency, n (%)
1q	20 (59)	6q	5 (15)
16p	17 (50)	8p	4 (12)
17q	15 (44)	18	4 (12)
8q	13 (38)	4q	3 (9)
11q	11 (32)	x	3 (9)
20q	11 (32)	17p	3 (9)
1p	8 (24)		
20p	8 (24)		
19q	7 (21)		
19p	6 (18)		

## References

[1] Kallioniemi A, Kallioniemi OP, Sudar D (1992). Comparative genomic hybridization for molecular cytogenetic analysis of solid tumors. Science.

[2] Weiss MM, Hermsen MA, Meijer GA (1999). Comparative genomic hybridisation. Mol Pathol.

[3] Pinkel D, Albertson DG (2005). Array comparative genomic hybridization and its applications in cancer. Nat Genet.

[4] Forozan F, Karhu R, Kononen J, Kallioniemi A, Kallioniemi OP (1997). Genome screening by comparative genomic hybridization. Trends Genet.

[5] Oostlander AE, Meijer GA, Ylstra B (2004). Microarray-based comparative genomic hybridization and its applications in human genetics. Clin Genet.

[6] Yang L, Parkin DM, Ferlay J, Li L, Chen Y (2005). Estimates of cancer incidence in China for 2000 and projections for 2005. Cancer Epidemiol Biomarkers Prev.

[7] Stratton MR, Rahman N (2008). The emerging landscape of breast cancer susceptibility. Nat Genet.

[8] Kallioniemi A, Kallioniemi OP, Piper J, Tanner M, Stokke T, Chen L, Smith HS, Pinkel D, Gray JW, Waldman FM (1994). Detection and mapping of amplified DNA sequences in breast cancer by comparative genomic hybridization. Proc Natl Acad Sci USA.

[9] Abulhasan SJ, Tayel SM, Al-Awadi SA (1999). Mosaic Turner syndrome: cytogenetics versus FISH. Ann Hum Genet 63 (Pt 3).

[10] Wei F, Ni J, Wu SS, Liu H, Xu X, Han YL, Cai Y, Zhang JW, Chen XJ, Pang H (2002). Cytogenetic studies of esophageal squamous cell carcinomas in the northern Chinese population by comparative genomic hybridization. Cancer Genet Cytogenet.

[11] du Manoir S, Schröck E, Bentz M, Speicher MR, Joos S, Ried T, Lichter P, Cremer T (1995). Quantitative analysis of comparative genomic hybridization. Cytometry.

[12] Stacher E, Boldt V, Leibl S, Halbwedl I, Popper HH, Ullmann R, Tavassoli FA, Moinfar F (2011). Chromosomal aberrations as detected by array comparative genomic hybridization in early low-grade intraepithelial neoplasias of the breast. Histopathology.

[13] Yang XR, Killian JK, Hammond S (2015). Characterization of genomic alterations in radiation-associated breast cancer among childhood cancer survivors, using comparative genomic hybridization (CGH) arrays. PLoS One.

[14] Bonnet F, Guedj M, Jones N (2012). An array CGH based genomic instability index (G2I) is predictive of clinical outcome in breast cancer and reveals a subset of tumors without lymph node involvement but with poor prognosis. BMC Med Genomics.

[15] Arnedos M, Scott V, Job B (2012). Array CGH and PIK3CA/AKT1 mutations to drive patients to specific targeted agents: a clinical experience in 108 patients with metastatic breast cancer. Eur J Cancer.

[16] du Manoir S, Speicher MR, Joos S, Schröck E, Popp S, Döhner H, Kovacs G, Robert-Nicoud M, Lichter P, Cremer T (1993). Detection of complete and partial chromosome gains and losses by comparative genomic in situ hybridization. Hum Genet.

[17] Kim SW, Kim JW, Kim YT, Kim JH, Kim S, Yoon BS, Nam EJ, Kim HY (2007). Analysis of chromosomal changes in serous ovarian carcinoma using high-resolution array comparative genomic hybridization: Potential predictive markers of chemoresistant disease. Genes Chromosomes Cancer.

[18] Corrêa NC, Kuasne H, Faria JA, Seixas CC, Santos IG, Abreu FB, Nonogaki S, Rocha RM, Aparecida Borges Silva G, Gobbi H (2013). Genomic and phenotypic profiles of two Brazilian breast cancer cell lines derived from primary human tumors. Oncol Rep.

[19] Ross JS, Fletcher JA (1998). The HER-2/neu oncogene in breast cancer: Prognostic factor, predictive factor, and target for therapy. Stem Cells.

[20] Han W, Woo JH, Jeon YK, Yang SJ, Cho J, Ko E, Kim TY, Im SA, Oh DY, Park IA (2011). 17p12 deletion in breast cancer predicts resistance to neoadjuvant chemotherapy. Exp Ther Med.

[21] Al Kuraya K, Schraml P, Torhorst J, Tapia C, Zaharieva B, Novotny H, Spichtin H, Maurer R, Mirlacher M, Köchli O (2004). Prognostic relevance of gene amplifications and coamplifications in breast cancer. Cancer Res.

[22] Sun Q, Yang YM, Yu SH, Zhang YX, He XG, Sun SS, Liang XS, Pang D (2012). Covariation of copy number located at 16q22.1: New evidence in mammary ductal carcinoma. Oncol Rep.

[23] Larramendy ML, Lushnikova T, Björkqvist AM, Wistuba II, Virmani AK, Shivapurkar N, Gazdar AF, Knuutila S (2000). Comparative genomic hybridization reveals complex genetic changes in primary breast cancer tumors and their cell lines. Cancer Genet Cytogenet.

[24] Nishizaki T, DeVries S, Chew K (1997). Genetic alterations in primary breast cancers and their metastases: Direct comparison using modified comparative genomic hybridization. Genes Chromosomes Cancer.

[25] Nishizaki T, Chew K, Chu L, Isola J, Kallioniemi A, Weidner N, Waldman FM (1997). Genetic alterations in lobular breast cancer by comparative genomic hybridization. Int J Cancer.

[26] Yeatman TJ, Cantor AB, Smith TJ (1995). Tumor biology of infiltrating lobular carcinoma. Implications for management. Ann Surg.

[27] Richard F, PacynaGengelbach M, Schlüns K, Fleige B, Winzer KJ, Szymas J, Dietel M, Petersen I, Schwendel A (2000). Patterns of chromosomal imbalances in invasive breast cancer. Int J Cancer.

[28] MalamouMitsi VD, Syrrou M, Georgiou I, Pagoulatos G, Agnantis NJ (1999). Analysis of chromosomal aberrations in breast cancer by comparative genomic hybridization (CGH). Correlation with histoprognostic variables and c-erbB-2 immunoexpression. J Exp Clin Cancer Res.

[29] Fung LF, Wong N, Tang N, Lau A, Wong V, Pang CP, Suen M, King W, Johnson PJ (2001). Genetic imbalances in pT2 breast cancers of southern Chinese women. Cancer Genet Cytogenet.

[30] Mertens F, Johansson B, Höglund M, Mitelman F (1997). Chromosomal imbalance maps of malignant solid tumors: A cytogenetic survey of 3185 neoplasms. Cancer Res.

